# Benefits of Genomic Insights and CRISPR-Cas Signatures to Monitor Potential Pathogens across Drinking Water Production and Distribution Systems

**DOI:** 10.3389/fmicb.2017.02036

**Published:** 2017-10-19

**Authors:** Ya Zhang, Masaaki Kitajima, Andrew J. Whittle, Wen-Tso Liu

**Affiliations:** ^1^Department of Civil and Environmental Engineering, University of Illinois at Urbana-Champaign, Urbana, IL, United States; ^2^Division of Environmental Engineering, Faculty of Engineering, Hokkaido University, Sapporo, Japan; ^3^Department of Civil and Environmental Engineering, Massachusetts Institute of Technology, Cambridge, MA, United States

**Keywords:** virulence, genomic analysis, drinking water distribution systems, *Legionella*, *Mycobacterium*, *Parachlamydia*, *Leptospira*, CRISPR

## Abstract

The occurrence of pathogenic bacteria in drinking water distribution systems (DWDSs) is a major health concern, and our current understanding is mostly related to pathogenic species such as *Legionella pneumophila* and *Mycobacterium avium* but not to bacterial species closely related to them. In this study, genomic-based approaches were used to characterize pathogen-related species in relation to their abundance, diversity, potential pathogenicity, genetic exchange, and distribution across an urban drinking water system. Nine draft genomes recovered from 10 metagenomes were identified as *Legionella* (4 draft genomes), *Mycobacterium* (3 draft genomes), *Parachlamydia* (1 draft genome), and *Leptospira* (1 draft genome). The pathogenicity potential of these genomes was examined by the presence/absence of virulence machinery, including genes belonging to Type III, IV, and VII secretion systems and their effectors. Several virulence factors known to pathogenic species were detected with these retrieved draft genomes except the *Leptospira*-related genome. Identical clustered regularly interspaced short palindromic repeats-CRISPR-associated proteins (CRISPR-Cas) genetic signatures were observed in two draft genomes recovered at different stages of the studied system, suggesting that the spacers in CRISPR-Cas could potentially be used as a biomarker in the monitoring of *Legionella* related strains at an evolutionary scale of several years across different drinking water production and distribution systems. Overall, metagenomics approach was an effective and complementary tool of culturing techniques to gain insights into the pathogenic characteristics and the CRISPR-Cas signatures of pathogen-related species in DWDSs.

## Introduction

Over 500 waterborne or water-based pathogens of potential concern in drinking water (e.g., *Legionella pneumophila, Escherichia coli* O157:H7, *Mycobacterium avium*, and *Cryptosporidium parvum*) have been included in the Candidate Contaminant List by the US Environmental Protection Agency (EPA; Ashbolt, 2015). The traditional approach to identify these pathogens is through cultivation and then biochemical/serological tests or 16S rRNA gene-based phylogeny analysis (Lye and Dufour, 1993; Edberg et al., 1996; Stelma et al., 2004). However, identifying pathogens at species level does not always translate into health risks as some strains of the same species are more pathogenic than others (Schmidt and Schaechter, 2012).

Alternatively, comparative genomic analysis has become an effective way to evaluate the pathogenicity potential. It is reported that pathogens infect host through a multi-step process from entering the host, adhering to host tissues, penetrating or evading host defenses, damaging host tissues, to exiting the host. As a result, various virulence factors (VFs) are required for pathogenic species during the infection process, which can be divided into several general groups based on the conservation of similar mechanisms, such as adhesins, invasins, toxins, protein secretion systems, and antibiotic resistance mechanisms (Finlay and Falkow, 1997; Wilson et al., 2002). Thus, the presence of a set of virulence machinery in a bacterial genome has been used to define pathogenic subpopulations (Chapman et al., 2006; Cazalet et al., 2008; Bouzid et al., 2013; Foley et al., 2013; Picardeau, 2017). The knowledge on virulence machinery and the functions of key VFs in the literature have facilitated the usage of virulence machinery to evaluate health risks associated with pathogens in drinking water distribution systems (DWDSs; Wu et al., 2008; Huang et al., 2014). Secretion systems are essential for the transportation of proteins (i.e., effectors) from the cytoplasm into host cells or host environments to enhance attachment to eukaryotic cells, scavenge resources in an environmental niche, and disrupt target cell functions (Green and Mecsas, 2016). Some secretion systems are dedicated for bacteria-host interaction, such as the type III secretion system (T3SS) in *Chlamydia* (Betts-Hampikian and Fields, 2010), the type IVB secretion system (T4BSS, Dot/Icm) in *Lg. pneumophila* (Voth et al., 2012), and the type VII secretion system (T7SS) in *Mycobacterium* (Costa et al., 2015). The deletion of these secretion systems could result in a substantial decrease in virulence (Costa et al., 2015). In addition, several other VFs have also been reported for pathogens including those facilitating attachment and invasion (e.g., cell wall, type IV pili) and endotoxins (i.e., lipopolysaccharides, LPS; Schroeder et al., 2010; Favrot et al., 2013; Tortora et al., 2013.

While the identification of pathogens of potential concern in DWDSs is an important task, recent studies have often detected pathogens simultaneously together with their closely related species, which are often present at higher abundance. These include, for example, *Lg. pneumophila*-related species such as *Lg. dumoffii* (Hsu et al., 1984)*, Lg. sainthelensis* (Rodriguez-Martinez et al., 2015), and *Lg*. *jordanis* (Hsu et al., 1984; Kao et al., 2014), and *M. avium*-related species such as *Mycobacterium gordonae* (Falkinham et al., 2001; Lalande et al., 2001; Vaerewijck et al., 2005), *Mycobacterium immunogenum* (Gomez-Alvarez and Revetta, 2016a), and *Mycobacterium chelonae* (Gomez-Alvarez and Revetta, 2016b). Some of these species have been associated with illness and infections in clinical environments, including *Lg. dumoffii* (Yu et al., 2002), *M. gordonae* (Lalande et al., 2001)*, M. immunogenum* (Wilson et al., 2001), and *M. chelonae* (Lowry et al., 1990). As pathogens and their closely related species often share ecological niches (predominantly in biofilms), genetic exchange through conjugation and transformation occurs between the two groups, sometimes involving VFs (Gimenez et al., 2011; Gomez-Valero et al., 2011). However, it is not clear whether they possess similar VFs as observed in pathogens.

Furthermore, in DWDSs, pathogens and their closely related species mostly reside within biofilms where protozoa predation and viral lysis occur more frequently than bulk water, and have developed mechanisms to resist predation by inhibiting phagosome acidification and lysosome fusion of protozoa (Hilbi et al., 2001; Tilney et al., 2001). Phage DNA can be integrated into bacterial genomes by horizontal gene transfer as prophages, which are major contributors to differences among individuals within a bacterial species (Bobay et al., 2014). To protect bacteria from phage lysis, encountered foreign DNA fragments can be integrated into a clustered regularly interspaced short palindromic repeats-CRISPR-associated proteins (CRISPR-Cas) locus as spacers (Makarova et al., 2015). Through addition of spacers at one end of the CRISPR array and conservation of spacers at the other end (the leader distal end), the CRISPR-Cas system participates in a constant evolutionary battle between phages and bacteria (Deveau et al., 2010; Sun et al., 2016). This mechanism has been used as a vital tool for strain typing in epidemiology for the recognition of outbreaks and identification of infection sources (Horvath et al., 2008; Shariat and Dudley, 2014). Nevertheless, it is not clear how intracellular growth and phage integration might impact the genomic composition and virulence of pathogen-related species.

In this study, metagenomics analysis instead of cultivation based methods was carried out to investigate virulence machinery and genomic signatures as the result of phage integration in pathogens-related species in a drinking water production and distribution system. A groundwater-derived drinking water system studied previously (Ling et al., 2016; Zhang et al., 2017) was used as a model system. It consists of abstraction, softening, recarbonation, disinfection, filtration, and final distribution with a disinfectant residual (free chlorine). Samples of microbial biomass from 10 locations of the water production process and the distribution system were collected and community metagenomes sequenced (Zhang et al., 2017). Coupling digital droplet PCR (ddPCR) with metagenomics, draft genomes affiliated with known pathogen genera were recovered to reveal their abundance, diversity, potential pathogenicity, genetic exchange, and distribution across an urban drinking water system.

## Materials and methods

### Sampling and DNA extraction

Microbial biomass samples from different stages of the treatment processes and different locations in the distribution system were collected from a groundwater-sourced drinking water system. Detailed description of the studied drinking water system can be found in a previous study (Zhang et al., 2017) and in Figure S1. Briefly, these samples were from raw water (RW), immediately before filtration and chlorination (BC), finished water (FW) prior to distribution, three taps (DS1-DS3), two retired water mains (PB1-PB2), 14 household water meters (WM, combined into one sample), and five premise plumbing pipe reactors (PR, combined into one sample). The three tap water sampling sites (DS1-3) were located approximately one mile apart from each other to represent different locations within the DWDS. For water-phase samples (including RW, BC, FW, and DS1-3), a 10-min flushing (the cold-water side) was carried out before each sampling event to minimize the influence of premise plumbing before installing point-of-use water purifiers (Toray Industries Inc. Japan). Approximately 2,000 L of water was filtered during each sampling event at each site over a time span of 48 hrs. Water purifiers were collected at the end of each sampling event and transported to the laboratory in cools (the Department of Civil and Environmental Engineering, University of Illinois at Urbana-Champaign). They were disassembled after arriving at the laboratory and cells were washed off from the multilayer hollow fiber membrane with phosphate-buffered saline (PBS) through sonication (Symphony™ Ultrasonic Cleaners, VWR). The obtained mixture was filtered through 0.22 μm membranes and the membranes with cells were stored at −80°C. To obtain a better representation of the average composition, water-phase sampling was repeated four times, in June, July, August, and September 2014, except the BC sample due to membrane blockage (Zhang et al., 2017).

For biofilm samples, PB1 was a 2.25-inch cast iron water main installed in 1968 and PB2 was a 1.5-inch cast iron water main installed prior to 1927. Each pipe was cut into two 12-inch long pieces on site with an effort to minimize contamination. Additionally, 14 water meters were obtained through the local drinking water plant. For the PR sample, five galvanized pipes of the plumbing system of a dormitory were obtained within the service area of the studied system, which were installed before World War II (size = 2 inch, OD = 2.375 inch, ID = 2.067 inch, length = 14 feet). Detailed description and handling of these samples could be found in our previous study (Zhang et al., 2017). The biofilm samples were swabbed off the surfaces, re-suspended in PBS, and collected by filtering through 0.22 μm membranes. All the membranes with cells were stored at −80°C. Genomic DNA (gDNA) was extracted using FastDNA® SPIN Kit for Soil (MP Biomedicals, Carlsbad, CA, USA) from these membranes with cells following manufacturer's protocol with an elution volume of 50 μl. The effect of different DNA extraction methods on the quantity and quality of DNA yields from drinking water biofilms had been evaluated and published in a previous study (Hwang et al., 2012).

### ddPCR and real-time PCR

ddPCR was used to quantify total *Bacteria* and *Archaea* 16S rRNA genes and pathogens of potential concern, including *Mycobacterium* spp., *M. tuberculosis* complex, *Legionella* spp., *Lg. pneumophila, Pseudomonas aeruginosa*, and *Aeromonas hydrophila*, in the combined samples submitted for metagenomic sequencing, except DS1 and DS3 due to not enough gDNA. TaqMan-based ddPCR assays using primer/probe sets specific to each target (Table S1) were performed with a QX200™ Droplet Digital™ PCR System using ddPCR™ Supermix for Probes (Bio-Rad, Pleasanton, CA, USA). In addition, three eukaryotic groups (amoebae), *Naegleria fowleri, Acanthamoeba* spp., and *Balamuthia madrillaris*, were tested with TaqMan-based real-time PCR assays using primer/probe sets specific to internal transcribed spacer (ITS)/18S rRNA gene of each target (Table S1). Real-time PCR was performed with a CFX96™ Real-Time PCR Detection System using SsoAdvanced™ Universal Probes Supermix (Bio-Rad, Pleasanton, CA, USA). Because of the large variations in the number of ITS/18S rRNA genes in different eukaryotic species, only cycle threshold (*C*_*T*_) values were reported. Positive control (standard plasmid DNA) and negative control (H_2_O) were included in every ddPCR and real-time PCR reaction to ensure the successful amplification and the absence of contamination, respectively.

### Amplicon sequencing and metagenome sequencing analyses

16S rRNA gene amplicon analysis was carried out using a universal primer set targeting the V4-V5 hypervariable regions of both the Bacteria and Archaea domains (515F: 5′-GTGCCAGCMGCCGCGGTAA-3′ and 909R: 5′-CCCGTCAATTCMTTTRAGT-3′) using the Illumina Miseq platform with dual indexing strategy as described in a previous study (Zhang et al., 2017). DNA libraries for metagenomic sequencing were prepared by combining all the extracted gDNA from each sampling site due to the requirement of a relatively large amount of gDNA (>0.1 μg). The prepared library was paired-end sequenced on Illumina HiSeq2500 platforms (Illumina, Inc., San Diego, CA, USA) as described previously (Zhang et al., 2017).

### 16S rRNA gene sequencing analysis

The obtained paired-end 16S rRNA gene sequences were aligned with Mothur (Kozich et al., 2013). The resulting sequences were screened for chimeras by the UCHIME algorithm implemented in USEARCH 6.1 and processed using the *de novo* OTU picking workflow in QIIME as described previously (Zhang et al., 2017). EMIRGE was used to reconstruct nearly full-length SSU genes in metagenomes (Miller, 2013).

### Draft genome reconstruction

**Figure 1:**
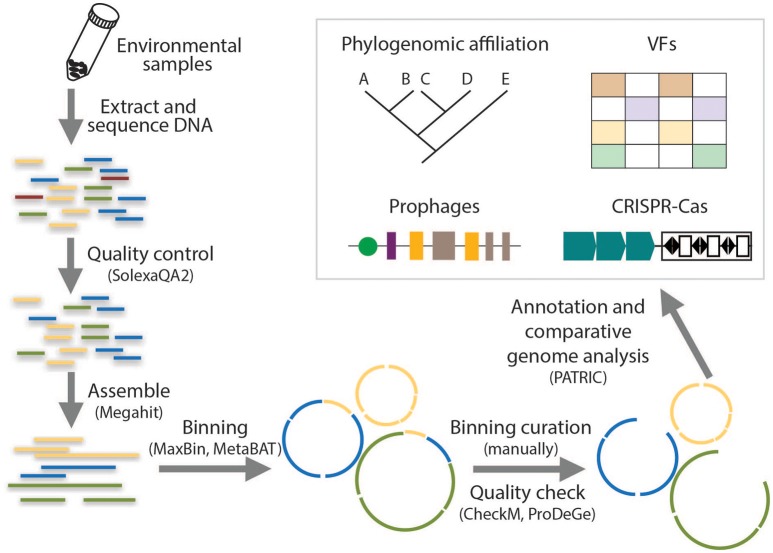
Flowing chart on the analysis of metagenomic data for the reconstruction of draft genomes and the identification of genetic signatures.

Draft genomes are presented as a set of sequence fragments or contigs, which are the most common form of genome assemblies obtained using metagenomics sequencing binning pipelines and account for two thirds of the bacterial genomes available in the GenBank database (Nagarajan et al., 2010; Edwards and Holt, 2013). Figure 1 illustrates the workflow of draft genome recovery used in this study. All the metagenomic datasets were trimmed using SolexaQA2 based on a cutoff of 20 by phred scores (Cox et al., 2010) and assembled using Megahit (Li et al., 2015). High-quality contigs (~2.0 × 10^8^ bp for each metagenome) were obtained at this step, to which >85.0% of the raw reads could be mapped except the RW sample. The longest contig in each metagenome was >4.0 × 10^5^ bp. More details of the assemblies could be found in our previous study (Zhang et al., 2017). The obtained contigs were binned based on metagenomics read coverage, tetranucleotide frequency, and the occurrence of unique marker genes by using both MaxBin 2.0 (Wu et al., 2016) and MetaBAT (Kang et al., 2015) to minimize the contamination of each bin. These two binning methods employed different clustering methods for the determination of different bins: MaxBin compares the distributions of distances between and within the same bins whereas MetaBAT clusters contigs iteratively by modified K-methods algorithm. Bins of pathogen-related species from the two binning tools were compared and assessed with CheckM (Parks et al., 2015) and ProDeGe (Tennessen et al., 2016), followed by manual curation. The curated bins with ≥90% completeness and ≥15-fold coverage were finalized as draft genomes. Details of each step in the pipeline had been reviewed and summarized by Sangwan et al. (2016) and a step-by-step tutorial of the workflow supplied with a sample dataset had been available by Edwards and Holt (2013). Percentages of reads mapped over the refined genome bins were estimated by Burrow-Wheeler Aligner-mem (Li and Durbin, 2009). The entire workflow was computed on a high-performance workstation (DELL precision T7600) equipped with 136 GB memory.

### Identification of VFs

Draft genomes of pathogen-related species retrieved were uploaded into PATRIC for annotation and feature identification (Wattam et al., 2014). VFs of different pathogens were collected from the literature and the VF database (VFDB, http://www.mgc.ac.cn/VFs/; Chen et al., 2012). Reported virulence genes within *Lg. pneumophila* included: the type II secretion system (T2SS, Lsp) for growth at low temperatures (Soderberg et al., 2008); the T4ASS (Lvh, F-type, and P-type) associated with conjugal DNA transfer and potentially in virulence (Gomez-Valero et al., 2011); the T4BSS (Dot/Icm) translocating several hundred effector proteins to support intracellular growth (Burstein et al., 2016); T4BSS-type effectors such as *ralF, lidA, sdhA*, and *lepAB* genes (Newton et al., 2010); type IV pili (*pilB,C,D*) involving in the entry to host cells, biofilm development, formation, type II protein secretion, and horizontal gene transfer (Schroeder et al., 2010); LPS transport (Lpt) proteins; and *mip* (macrophage infectivity potentiator) gene associated with the ability of *Lg. pneumophila* to replicate in eukaryotic cells (Newton et al., 2010).

For *M. tuberculosis*, the reported VFs included: the T7SS, also known as the ESX pathway (ESX-1 to ESX-5) to secrete proteins across their complex cell envelope (Houben et al., 2014); early secretory antigenic target (ESAT6), *esxA, H*, and *N*; culture filtrate protein-10 kDa (CFP-10), *esxB, G*, and *M* (Li et al., 2005); *pe*/*ppe* genes unique to mycobacteria and abundant in pathogenic mycobacteria (Sampson, 2011); antigen 85 (*ag85*) complex and mycolic acid cyclopropane synthase (*pcaA*) required for the biosynthesis of major components of the cell envelope (Favrot et al., 2013); adhesin (*hbhA*); phospholipase C (*plcC*); and oxidative stress reducer (*ahpC*; Forrellad et al., 2013).

For leptospires, some potential VFs identified in the literature included: *lipL32, mce, invA, atsE, mviN, rfb* for attachment and invasion and *asd, trpE*, and *sphH* for amino acid biosynthesis (Ren et al., 2003; Ko et al., 2009; Fouts et al., 2016).

For *Parachlamydia*, known VFs included: negative regulator of the T3SS, SctW; protein kinase, Pkn5; translocated actin-recruiting phosphoprotein, *tarp*; inclusion membrane proteins IncA to IncG; translocator protein, CopB; modulation of host cell apoptosis, CADD; and Mip (Greub, 2009; Betts-Hampikian and Fields, 2010; Collingro et al., 2011; Croxatto et al., 2013). Furthermore, genes coding for nucleotide transporters that import host cell ATP in exchange for ADP (*ntt*) were part of the complex involving in bacteria-host interaction, but were generally not considered as VFs (Schmitz-Esser et al., 2004; Haferkamp et al., 2006).

### Construction of phylogenomic tree

PhyloPhlAn (Segata et al., 2013) was used to construct phylogenomic trees based on draft genomes and reference genomes. The constructed trees were visualized using iTOL (Letunic and Bork, 2016).

### Identification of antibiotic resistance genes (ARGs) and CRISPR-Cas loci

ARGs and CRISPR-Cas regions were screened with PATRIC. The identified CRISPR loci and ARGs were confirmed with CRISPRfinder (Grissa et al., 2007) and ResFinder (Zankari et al., 2012), respectively. Identified CRIPSR-Cas loci were classified into the current system consisting of two classes, five types, and 16 subtypes (type I-A to I-F and I-U, type II-A to II-C, type III-A to III-D, type IV, and type V) based on *cas* genes and additional signature genes (Makarova et al., 2015). Additionally, we investigated the possible targets (protospacers) of spacers in CRISPR-Cas arrays within the obtained draft genomes using CRISPRTarget to search against all the available databases (i.e., GenBank-Phage, GenBank-Environmental, RefSeq-Plasmid, RefSeq-Viral, and RefSeq-Bacteria), which was combined with the known features of each subtype that had been reported to be essential for target recognition, such as protospacer adjacent motifs (PAMs) and seed regions (Biswas et al., 2013). Extra weighting was given to known PAMs: 5′-GG-3′ for I-F (Mojica et al., 2009) at the 3′ region of protospacer and 5′-CCN-3′ for II-B (Fonfara et al., 2014) at the 5′ region of protospacer. Moreover, we also manually examined seed sequences (8-nt for Type I-F and 13-nt for Type II-B) within the match. PHAST was used to identify prophage sequences in these draft genomes (Zhou et al., 2011).

### Genomic data depositing

The nine draft genomes reconstructed in this study are deposited in GenBank under the BioProject PRJNA323575 with BioSamples SAMN07572181- SAMN07572189.

## Results

### Detection of pathogens of potential concern in the system

**Figure 2:**
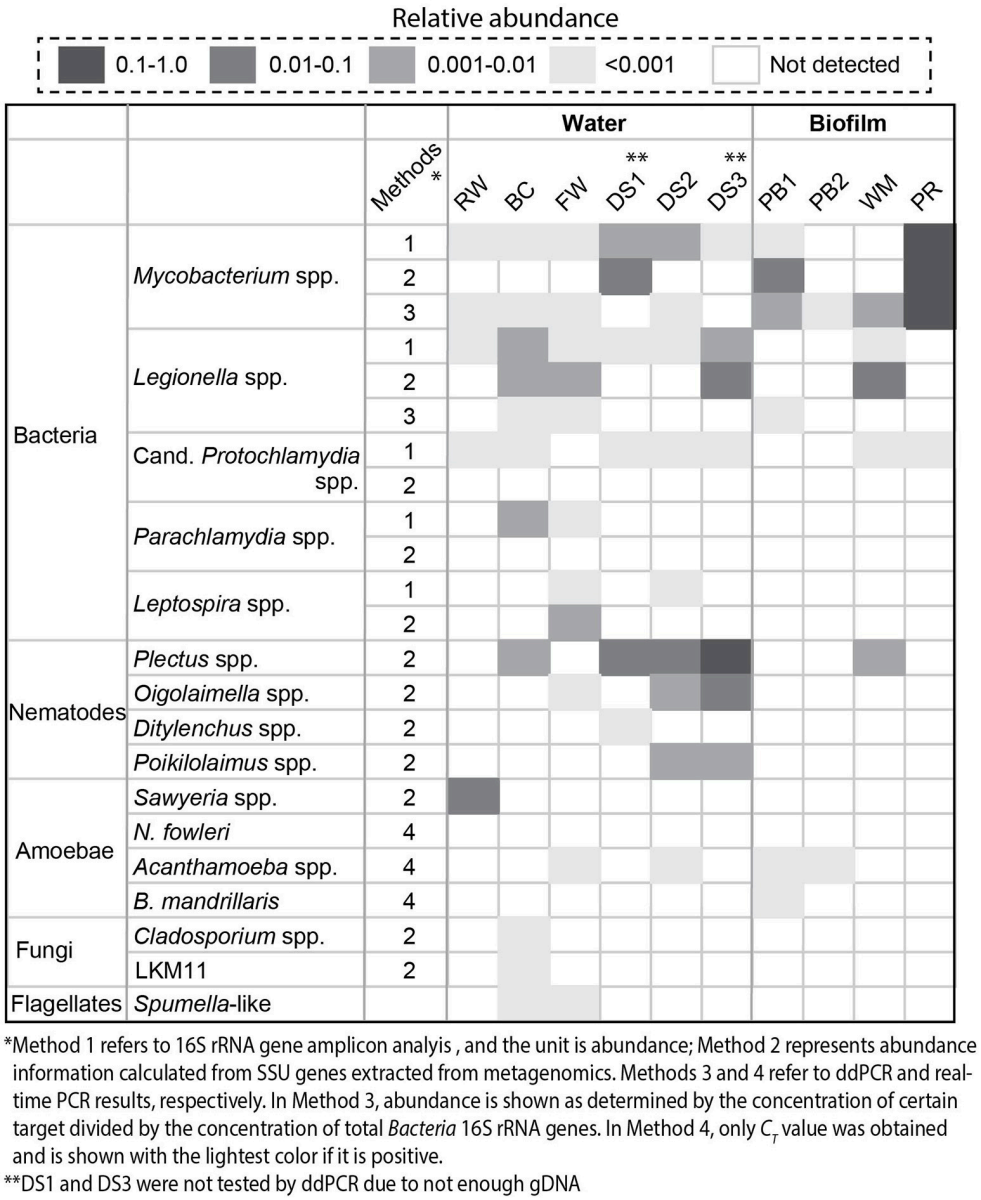
Detected potential pathogens and eukaryotes (nematodes, amoebae, fungi, and flagellates) by 16S rRNA gene amplicon analysis (1), SSU genes extracted from metagenomes (2), ddPCR (3), and real-time PCR (4). Here, relative abundance was reported. The concentration of specific pathogens determined with ddPCR was summarized in Table S2. We divided the samples into water and biofilm phases.

A combination of different molecular biological techniques, namely, 16S rRNA gene amplicon sequencing, metagenomics, and ddPCR/real-time PCR was employed to investigate the diversity and quantity of potential pathogens in the drinking water production and distribution system. Regarding prokaryotes, Figure 2 shows that in general, the distribution system samples contained the highest relative abundance of *Mycobacterium* spp. and *Legionella* spp. in comparison with samples from the treatment process. The highest level of *Mycobacterium* spp. was detected with the PR sample with a relative abundance of 1.3 × 10^−1^ and an absolute concentration of 3.3 × 10^4^ copies/ng-gDNA by ddPCR (Table S2). The BC sample contained the highest level of *Legionella* spp.: a relative abundance of 4.7 × 10^−3^ based on 16S rRNA amplicon analysis and a concentration of 40.9 copies/ng-gDNA by ddPCR. Despite the occurrence of potential pathogens at the genus level, known pathogenic species, including *M. tuberculosis* complex, *Lg. pneumophila*, and *A. hydrophila* were not detected (Table S2). Additionally, sequences related to *Candidatus* Protochlamydia spp., *Parachlamydia* spp., and *Leptospira* spp. were also detected (Figure 2). *Candidatus Protochlamydia* spp. and *Parachlamydia* spp. were endosymbionts of amoeba and emerging agents of pneumonia (Greub, 2009). Notably, *Candidatus Protochlamydia* spp. were detected in all the distribution water phase samples.

Meanwhile, we could identify various eukaryotes, such as nematodes, amoebae, and flagellates with metagenomics and real-time PCR that co-existed with these potential pathogens. *Plectus* spp. were the most abundant nematodes detected in the system and present in half of the samples. For amoebae, *Acanthamoeba* spp. were observed in FW, DS2, PB1, and PB2 while *Sawyeria* spp. were only found in RW.

### Characterization of pathogen-related species through the construction of draft genomes

**Figure 3:**
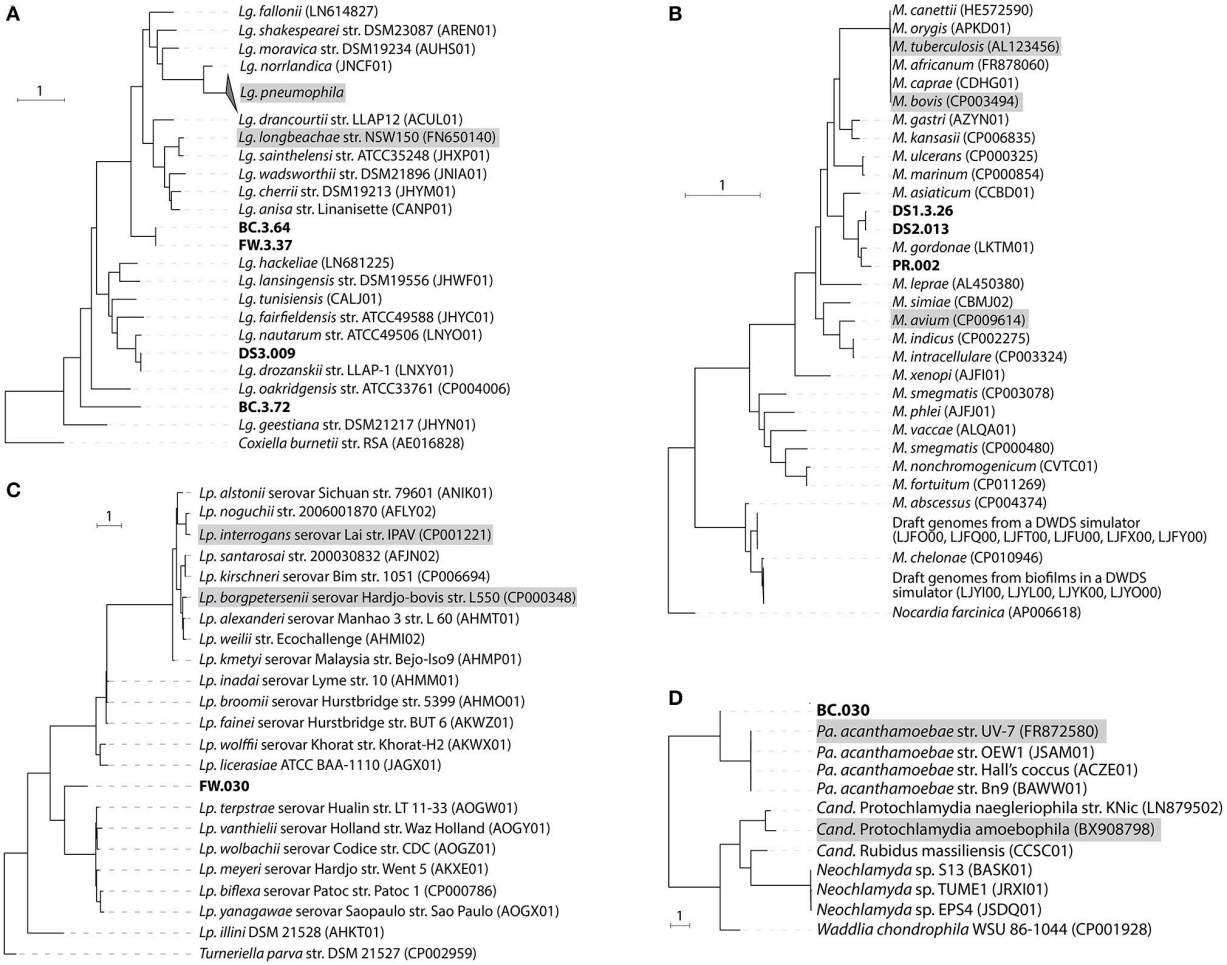
Phylogenomic tree of recovered draft genomes constructed based on up to 400 conserved protein sequences. **(A)** *Legionella*; **(B)** *Mycobacterium*; **(C)** *Leptospira*; **(D)** *Parachlamydia*. The nine draft genomes recovered from this study were bold. Known pathogenic species were shaded with gray. Scale bar, 1 expected substitutions per site.

Nine draft genomes closely related to known pathogens were successfully recovered from the metagenomes of BC, FW, DS1-3, and PR with ≥90% completeness and ≥15-fold coverage (Table 1). The phylogenomic tree in Figure 3 showed that four draft genomes were affiliated with *Legionella* (BC.3.64, FW.3.37, DS3.009, BC.3.72; Figure 3A), three with *Mycobacterium* (DS1.3.26, DS2.013, PR.002; Figure 3B), one with *Leptospira* (FW.030; Figure 3C), and one with *Parachlamydia* (BC.030; Figure 3D). In Figure 3A, different species of *Legionella* were observed to co-exist in the same niche, i.e., BC.3.64 and BC.3.72 in the BC sample. FW.3.37 was observed to be 99.7% similar to BC.3.64 in the average nucleotide identity (ANI) based on 400 marker genes. These three draft genomes probably represented new species of *Legionella* as they did not cluster together with any known species. A fourth draft genome, DS3.009, was affiliated with *Lg. drozanskii*. For *Mycobacterium* draft genomes, all three (DS1.3.26, DS2.013, PR.002) were closely related to *M. gordonae*. The *Leptospira* draft genome FW.030 was outside of the cluster containing mostly saprophytic species. Last, draft genome BC.030 fell between *Pa. acanthamoebae* and *Candidatus* Protochlamydia amoebophila. Collectively, five of the draft genomes retrieved were not closely related to any known isolated species, possibly due to the limitation of cultivation methods to recover microorganisms from drinking water systems so far.

**Table 1:** General features of the recovered genomes of pathogen-related species.

**Bin ID**	**Source**	**Affiliation**	**Completeness**	**Coverage**	**No. of contigs**	**Genome size (bp)**	**G+C content (%)**	**No. of protein-coding genes**	**Possibly missing genes**	**Median sequence size**	**Longest contig size**
BC.3.64	BC	*Legionella* sp.	94.44	30.13	62	2.27E+06	40.1	2112	5	31,419	150,921
BC.3.72	BC	*Legionella* sp.	94.51	23.78	22	1.95E+06	40.6	1829	11	74,242	336,208
FW.3.37	FW	*Legionella* sp.	94.15	27.68	63	2.10E+06	40.3	1926	14	18,840	221,613
DS3.009	DS3	*Legionella* sp.	98.83	45.78	140	3.36E+06	39.4	3159	39	16,314	165,891
DS1.3.26	DS1	*Mycobacterium* sp.	99.86	79.34	217	7.43E+06	66.8	6689	64	16,573	250,869
DS2.013	DS2	*Mycobacterium* sp.	99.86	23.74	219	7.96E+06	66.5	7334	77	15,428	244,689
PR.002	PR	*Mycobacterium* sp.	89.12	451.94	919	6.78E+06	67.0	6179	120	4,016	89,735
BC.030	BC	*Parachlamydia* sp.	100.00	24.81	39	3.04E+06	41.5	2763	15	54,962	289,998
FW.030	FW	*Leptospira* sp.	95.88	15.42	114	3.73E+06	35.1	3613	19	15,672	307,203

### VFs detected in the draft genomes recovered

**Figure 4:**
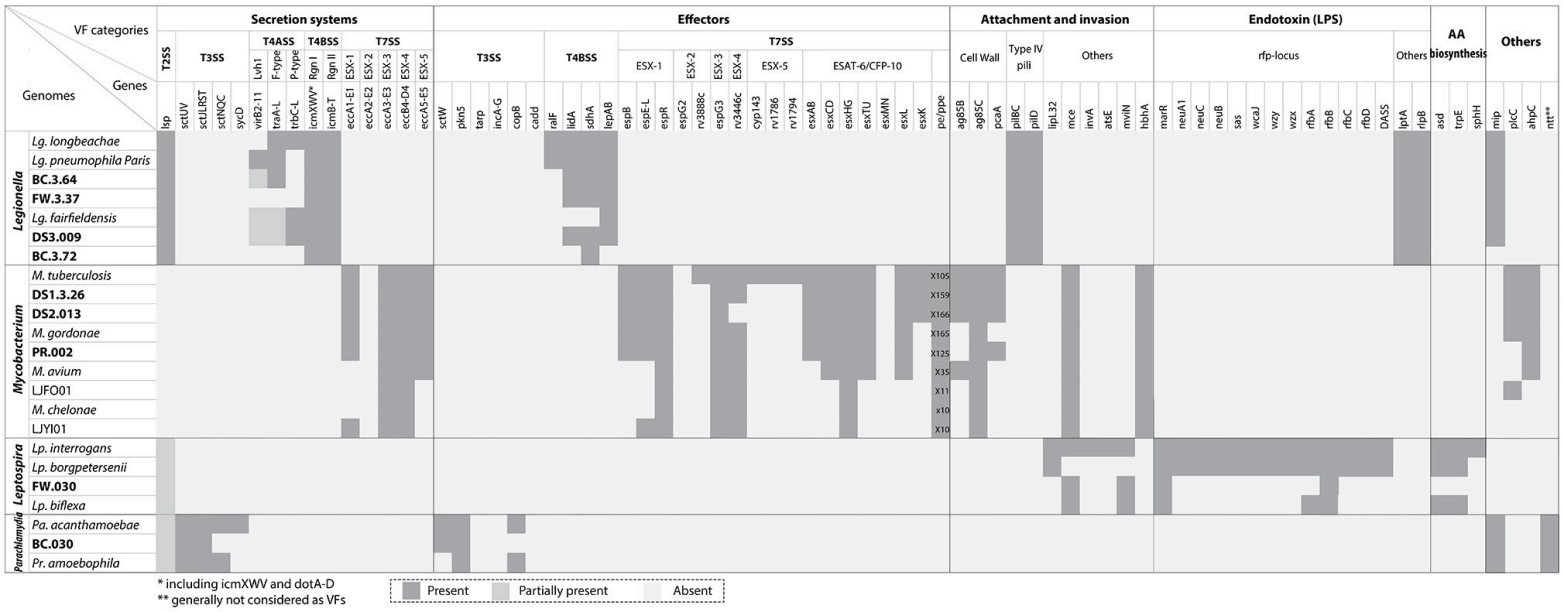
VFs identified with the draft genomes recovered in this study and related genomes from public databases. VFs were grouped based on general categories (secretion systems and associated effectors, attachment and invasion, endotoxin, amino acid biosynthesis, and others). The genomes were organized by their taxonomic affiliations. There were some shared VFs among different genera, including T2SS among *Legionella, Leptospira*, and *Parachlamydia*, the *mip* gene between *Legionella* and *Parachlamydia*, and the *mce* gene between *Mycobacterium* and *Leptospira*. The accession number of known genomes were listed in Table S3.

Figure 4 (also see Table S3) indicated the presence and absence of VFs affiliated with secretion systems, effectors, attachment and invasion, endotoxins (e.g., lipopolysaccharides), and amino acid biosynthesis found in the recovered draft genomes and their related reference genomes. For *Legionella* in the secretion system category, the T2SS and T4BSS were the major pathogenesis systems observed in all draft genomes recovered. By contrast, the T4ASS, associated with conjugal DNA transfer, was detected in BC.3.64 and DS3.009 but absent in BC.3.72 and FW.3.37 possibly due to non-existence in these bacteria or the inability or poor efficiency to retrieve and assemble sequences pertaining to these hypervariable regions (Pop, 2009; Gomez-Valero et al., 2011). In the effectors category, T4BSS-assicated VFs including *lidA, sdhA*, and *lepAB* genes but not *ralF* were detected in three of the four draft genomes. In addition, all draft genomes contained LPS transport related genes, *lptA* and *lptE*. Last, the *mip* gene was observed in BC.3.64, FW.3.37, and DS3.009, but not BC.3.72.

For *Mycobacterium*, ESX-1, ESX-3, and ESX-5 T7SSs were observed in all *Mycobacterium* draft genomes recovered. Effectors belonging to ESX-1 and ESX-3 could also be detected, including *esxAB* and *TU*, but not effectors belonging to ESX-5 (*cyp143, rv1786, rv1794*, and *esxMN*). For the *pe*/*ppe* multigene family, all the recovered draft genomes contained more than 100 such genes, which was comparable to those observed in pathogenic species. Other VFs detected included cell envelop biosynthesis, *ag85* (except in PR.002) and *pca*A; adhesin, *hbhA*; phospholipase C, *plcC*; and oxidative stress reducer, *ahpC*. For *Leptospira*, the known VFs were mainly associated with the attachment and invasion, endotoxin and amino acid biosynthesis categories, and among them four (i.e., *mce1B, mviN, marR*, and *rfbD*) were detected in FW.030. The T2SS was partially present in *Leptospira* spp., including FW.030, but the association of the T2SS with virulence had not been experimentally tested (Picardeau, 2017). For *Parachlamydia*, VFs were mainly observed in the T3SS and associated effector categories. Two VFs, the T2SS (partially) and *mip* in the “others” category were also observed. As *Parachlamdia* spp. and *Candidatus Protochlamydia* spp. were intracellular bacteria of amoebae like *Legionella* spp., they also possessed T2SSs and Mip systems. Five *ntt* genes were observed with BC.030, putatively belonging to three NTT isoforms (NTT1-3) as shown in Figure S2 (Haferkamp et al., 2006). Last, several ARGs related to the resistance of aminoglycoside (moderate level), beta-lactam, and chloramphenicol (antimicrobial peptides) could be detected in the *Legionella* draft genome DS3.009. All the *Mycobacterium* recovered draft genomes possessed the *aac(2*′*)-Ic* gene, which was universally distributed among all *Mycobacterium* spp. (Ainsa et al., 1997; Table S4).

### Usage of CRISPR-Cas signatures to monitor *Legionella* spp. across the studied system

**Figure 5:**
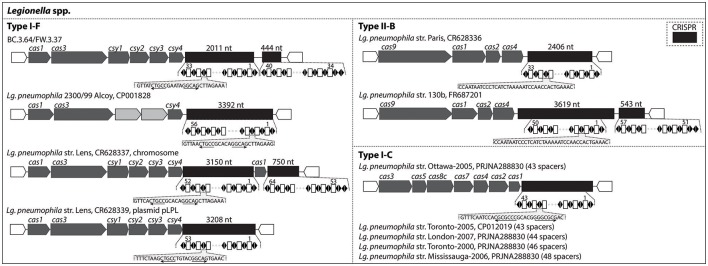
CRISPR-Cas loci identified in the draft genomes recovered in this study and known genomes of *Legionella*. They were organized according to the subtypes (Type I-F, II-B, and I-C) of CRISPR-Cas loci.

CRISPR-Cas genetic signatures, which are defense systems used by prokaryotes against viruses and not associated with pathogenicity, could be an effective tool to discriminate and monitor sub-lineages of pathogen-related species across the studied drinking water production and distribution system. Figure 5 indicates the type of CRISPR-Cas systems identified in the draft genomes recovered and in several published *Lg. pneumophila* genomes. Among the three known subtypes of *Lg. pneumophila* (I-F, II-B, and I-C), this study detected type I-F with BC.3.64 and FW.3.37 based on *cas* gene clusters. The type I-F CRISPR-Cas observed in these two draft genomes was almost identical, i.e., 99% sequence similarity for *cas1* gene and 100% sequence similarity for the remaining *cas* genes (Table S5). Together with the findings of phylogenomic classification and genome similarity (99.7%; Figure 3), BC.3.64 and FW.3.37 were very likely to belong to a closely-related population originated from the same ancestor traveling from upstream (BC) to downstream (FW) of the studied drinking water production and distribution system. There was not enough information to determine whether the strain was alive at the BC site or whether filtration and chlorination had inactivated the strain in FW. Their *cas* gene clusters shared relatively low protein sequence similarities (from <40–76%) with other type I-F CRISPR-Cas loci of *Lg. pneumophila* (Table S5). Last, a Type II-B CRISPR-Cas locus was detected with *Leptospira* draft genome FW.030 (Figure S3).

### Diversity of prophage

Table 2 shows the types of prophages found in the recovered draft genomes. Initially, 36 potential prophage sequences were identified using PHAST (Figure S4) and they were reduced to 16 by considering the presence of genes encoding integrases and/or cI-type repressors (Fan et al., 2014; Figure S5). The lengths of prophage regions varied from 9.5 to 40.1 kbp. Six were associated with *Legionella* draft genomes, seven with *Mycobacterium* draft genome, and one each with *Parachlamydia* and *Leptospira*. An intact prophage (37.1 kbp) was recovered from PR.002. Shared prophage structures were observed between BC.3.64 and FW.3.37 and between DS1.3.26 and DS2.013. In addition, DS2.013 contained as many as five prophage sequences, which was rare for *Mycobacterium* genomes. Last, a prophage region identified in FW.030 showed sequence similarities to *Pandoravirus salinus* which was the largest virus reported so far with genomes up to 2.5 Mb and restricted to *Acanthamoeba* as hosts (Philippe et al., 2013).

**Table 2:** Prophages identified in the retrieved draft genomes.

**Genera**	**Genomes**	**Regions**	**Length (kbp)**	**Possible phage**
*Legionella*	BC.3.64	R1	9.5	*Salisaeta* icosahedral phage 1
		R2	31.1	*Stenotrophomonas* phage S1
	FW.3.37	R1	9.5	*Salisaeta* icosahedral phage 1
		R2	26.1	*Caulobacter* virus Karma
	DS3.009	R1	37.0	*Stenotrophomonas* phage S1
		R2	23.5	*Haemophilus* phage HP2
*Mycobacterium*	DS1.3.26	R1	19.0	*Mycobacterium* phage Adler
	DS2.103	R1	28.3	*Mycobacterium* phage RhynO
		R2	12.2	Molluscum contagiosum virus subtype 1
		R3	27.7	*Mycobacterium* phage Adler
		R4	31.6	*Mycobacterium* phage Adler
		R5	40.1	*Mycobacterium* phage Adler
	PR.002	R1	17.2	*Mycobacterium* phage Adler
		R2	37.1	*Mycobacterium* phage Milly
*Leptospira*	FW.030	R1	29.9	*Pandoravirus salinus*
*Parachlamydia*	BC.030	R1	19.3	*Cronobacter* phage vB_CsaM_GAP32

## Discussion

### Potential virulence of pathogen-related species

Virulence machinery characterized by genomic analysis has been used to define pathogenicity for many known pathogens, such as *E. coli* (Chapman et al., 2006), *Salmonella* (Foley et al., 2013), *Cryptosporidium* (Bouzid et al., 2013), *Lg. pneumophila* (Cazalet et al., 2008), and *Leptospira* (Picardeau, 2017). This approach is used here to evaluate the potential pathogenicity of those draft genomes of pathogen-related species recovered from an urban drinking water system. *Legionella*-related draft genomes found at two different locations of the water production process (i.e., BC.3.64 and FW.3.37) shared almost identical genomic sequences and possessed almost all known VFs to *Lg. pneumophila* and *Lg. longbeachae*. Another strain found during the water production process (i.e., BC.3.72) was clustered outside of known pathogenic *Legionella* clusters, and possessed fewer virulence genes than the other three recovered strains (i.e., BC.3.64, FW.3.37, and DS3.009). While the finding that most of the draft genomes encoded a high number of VFs may raises concerns on their pathogenicity, previous studies on closely related species/strains of pathogenic *Aeromonas* found no correlations between the presence/absence of VFs and extraintestinal infections (Havelaar et al., 1992; Lye et al., 2007). Thus, further studies combining microbiological (e.g., cultivation and animal models), genomic, and metabolic (e.g., transcriptomics and proteomics) methods should be carried out to understand the role of these VFs at the level of gene expression, protein function and regulation, and interaction with host immune system to confirm the virulence of these strains for immunocompromised individuals. This framework, once established, can be transferred into a novel pathogen surveillance program that enables virulence assessment of a broad range of heterotrophic bacteria found in potable water to possibly identify currently unknown pathogens.

All three *Mycobacterium*-related draft genomes recovered were closely related to *M. gordonae*, which is less virulent than *M. tuberculosis*, but contained a high number of genes (over 100) related to *pe*/*ppe* and T7SS. In comparison, genomes of *M. immunogenum* (LJFO01) and *M. chelonae* (LJYI01) isolated from a chloraminated DWDS simulator in previous studies (Gomez-Alvarez and Revetta, 2016a,b) lacked ESX-1 or ESX-5 and contained fewer *pe/ppe* genes. Due to the prevalence of *M. gordonae* in tap water and biofilms, particularly in groundwater-derived drinking water systems (Vaerewijck et al., 2005), special attention to this group would be necessary. Pathogenic *Leptospira* are the causative agent of leptospirosis, which is the most widespread zoonotic disease infecting both human and animals (Evangelista and Coburn, 2010). In this study, the *Leptospira*-related genome FW.030 obtained did not contain most of the VFs known for *Lp. interrogans* and thus was likely not pathogenic. Among Parachlamydiaceae, only few strains such as *Pa. acanthamoebae* and *Candidatus Pr. naegleriophila* have been considered as emerging pathogens, causing mainly respiratory infections, while many others including *Neochlamydia hartmannellae* and *Pr. amoebophila* might be environmental strains or endosymbionts (Corsaro and Greub, 2006; Lamoth et al., 2011). Therefore, the pathogenic potential of *Parachlamydia*-related genome BC.030 remains to be determined.

### Use of spacers in CRISPR-Cas as biomarkers for *Legionella* subtyping

Due to the high genome plasticity of *Legionella* species, molecular typing by a single marker gene has been difficult. For instance, the *mip* gene is associated with the ability of *Lg. pneumophila* to replicate in eukaryotic cells, and has been extensively used as a biomarker to detect the presence/absence of *Lg. pneumophila* in a sample (Gomez-Valero et al., 2009). It was detected in three *Legionella*-related draft genomes constructed in this study: BC.3.64 and FW.3.37 were closely related to *Lg. fallonii*, and DS3.009 to *Lg. drozanskii* (Figure S6). However, the *mip* gene was limited in differentiating the *Lg. pneumophila* subspecies *fraseri* from other subspecies (Figure S4). Thus, the European Working Group for Legionella Infections (EWGLI) has suggested that a combination of several biomarkers, including *flaA, pilE, asd, mip, mompS, proA*, and *neuA*, should be used to effectively identify *Lg. pneumophila* (Fry et al., 2000; Gaia et al., 2005; Ratzow et al., 2007). However, phylogenetic incongruence (i.e., different lineages of the same strain indicated by different biomarkers) and limitations (i.e., the inability of some biomarkers to discriminate certain strains) in the discriminatory power of these multiple biomarkers could still occur because of differences in selection pressures associated with individual biomarkers.

Alternatively, spacers in CRISPR-Cas can be used as a biomarker in the monitoring of certain *Legionella* strains at an evolutionary scale of several years across drinking water production and distribution systems. The pattern of adding new spacers at one end of the CRISPR array and conserving spacers among common ancestors at the other end has been demonstrated with *Legionella* strains collected in Canada and Europe (CRISPR Type I-C and Type II-B; Ginevra et al., 2012; Lück et al., 2015; Rao et al., 2016). The longest time for these spacers to remain conserved among these strains and a *Leptospirillum* strain previously studied was reported to be 5 years or longer (Sun et al., 2016). As shown in Figure 5, Type I-F Cas loci were detected in the genomes of *Lg. pneumophila* str. 2300/99 Alcoy and str. Lens (both in the chromosome and plasmid). The two draft genomes recovered in our study, BC.3.64 and FW.3.37, also contained type I-F CRISPR-Cas loci, but the spacers were different from str. 2300/99 Alcoy and str. Lens. With 100% sequence similarity in CRISPR and high overall genomic similarity, these two genomes were likely derived from the same ancestor. Thus a specific CRISPR-Cas biomarker could be developed and used to monitor the distribution of this strain within the drinking water system studied. Furthermore, Types II-B and I-C were detected in a variety of *Lg. pneumophila* strains (Figure 5) and Type II-B was detected in 75.0% of the 400 *Lg. pneumophila* strains collected in a previous study (Ginevra et al., 2012). With more than 600 *Legionella* genomes available with NCBI's website and the diversity of CRISPR-Cas Types (I-C, I-F, and II-B) known among these strains, CRISPR-Cas spacers will be a promising biomarker for monitoring the distribution of *Legionella* at the strain level in samples taken from various drinking water systems, across different water bodies, and between patients over several years. However, cautions are needed when applying this method over a relatively large evolutionary scale as previous reports on *Yersinia pestis, Streptococcus thermophiles*, and *Leptospirillum* suggested that CRISPR loci could also evolve via internal deletion of spacers in the CRISPR array (Pourcel et al., 2005; Horvath et al., 2008; Sun et al., 2016).

### Origin of spacers in CRISPR-Cas of pathogen-related genomes

The interaction between bacteria and viruses in drinking water systems or, more broadly, in oligotrophic environments is not well understood (Lehtola et al., 2004; Liu et al., 2015; Guidi et al., 2016). Table 3 shows only 26 out of the 119 identified CRISPR-Cas spacers matched to entries in databases including GenBank-Phage, GenBank-Environmental, RefSeq-Plasmid, RefSeq-Viral, and RefSeq-Bacteria. Among them, 13 spacers matched sequences in other *Lg. pneumophila* strains. Two commonly observed targets were a 30-kb unstable genetic element previously identified in *Lg. pneumophila* str. RC1 and a 60-kb plasmid in *Lg. pneumophila* str. Lens. Likely, these elements were originated from bacteriophages in environments and incorporated into *Lg. pneumophila* genomes as mobile genetic elements such as prophages and plasmids. When the DNA of *Lg. pneumophila* was damaged or under other stress conditions, prophages could be excised, replicated, and ultimately used to lyse the host and spread into the environment. Ecologically, it would be rational for other *Lg. pneumophila* strains to incorporate their fragments into CRISPR systems so that they had the ability to destroy them when being attacked (Rao et al., 2016).

**Table 3:** Potential targets of CRISPR-Cas spacers in *Legionella*-related genomes.

**Genomes**	**Spacer ID**	**Hits for spacers**	**Score**	**Number of mismatches within the spacer**	PAMs^**^	**Seed sequence mismatch position**
BC.3.64	Sp6	Marine metagenome genome assembly TARA_030_DCM_0.22 (CENH01030675)	27	5	GG	8
Lgp^*^ Lens	Chrm_Sp23	*Lg. pneumophila* serogroup 1, 30 kb instable genetic element (AJ277755)	35	1	GG	6
	Chrm_Sp35	*Paenibacillus* sp. FSL H7-0357, complete genome (CP009241)	27	5	GG	3
	Plsm_Sp22	Activated sludge metagenome contig16020 (AERA01015926)	37	0	GG	–
	Plsm_Sp46	*Lg. pneumophila* serogroup 1, 30 kb instable genetic element (AJ277755)	35	1	GG	7
	Plsm_Sp12	*Lg. pneumophila* 2300/99 Alcoy, complete genome (NC_014125)	31	3	GG	7
	Plsm_Sp12	*Lg. pneumophila* str. Corby, complete genome (NC_009494)	31	3	GG	7
	Plsm_Sp10	*Lg. pneumophila* str. Paris complete genome (NC_006368)	30	1	Not match	N/A
	Plsm_Sp8	Uncultured marine Microviridae clone SOG3-01 major capsid protein gene, partial cds (KC131005)	29	4	GG	1
	Plsm_Sp47	Activated sludge metagenome contig16020 (AERA01015926)	29	4	GG	–
	Plsm_Sp50	Marine metagenome 1096626097875, whole genome shotgun sequence (AACY023989113)	29	4	GG	5
	Plsm_Sp7	Activated sludge metagenome contig06523 (AERA01006474)	29	5	GG	3, 5
	Plsm_Sp13	*Lg. pneumophila* 2300/99 Alcoy, complete genome (NC_014125)	26	3	Not match	N/A
	Plsm_Sp32	*Lg. pneumophila* str. Lens plasmid pLPL, complete sequence (NC_006366)	24	4	Not match	N/A
	Plsm_Sp7	*Lg. pneumophila* str. Lens plasmid pLPL, complete sequence (NC_006366)	24	4	Not match	N/A
Lgp Alcoy	Sp32	Uncultured Gokushovirinae clone WSBWG10n1 major capsid protein gene (KF689311)	31	3	GG	8
	Sp28	Marine metagenome genome assembly TARA_122_SRF_0.1-0.22 (CETN01079705)	29	4	GG	–
	Sp3	*Lg. pneumophila* str. Lens plasmid pLPL (NC_006366)	26	3	Not match	N/A
Lgp Paris	Sp33	Activated sludge metagenome contig28417 (AERA01027227)	37	3	CCA	6, 9
	Sp4	*Schistocephalus solidus* genome assembly S_solidus_NST_G2 (LL901847)	29	5	CCA	–
	Sp15	*Lg. pneumophila* str. Lens plasmid pLPL (NC_006366)	28	3	Not match	N/A
	Sp14	*Lg. pneumophila* 130b draft genome (FR687201)	28	4	Not match	N/A
Lgp 130b	Sp40	*Lg. pneumophila* str. Paris complete genome (NC_006368)	37	0	CCA	–
	Sp41	Hypersaline lake metagenome ctg7180000052828 (APHM01003927)	30	5	CCA	10
	Sp27	*Lg. pneumophila* str. Corby, complete genome (NC_009494)	30	2	Not match	N/A
	Sp27	*Lg. pneumophila* 2300/99 Alcoy chromosome (NC_014125)	30	2	Not match	N/A

* Lgp, Lg. pneumophila;

** *PAMs, protospacer adjacent motifs*.

We also observed near-perfect matches of four spacers in CRISPR-Cas to one activated sludge metagenome (AERA01; More et al., 2014). It has been reported that wastewater treatment plants (WWTPs) contained 10–1,000 times higher viral concentration than in natural aquatic environments, making WWTP an important reservoir and source of viruses (Edwards and Rohwer, 2005; Tamaki et al., 2012). In the studied drinking water production and distribution system, we estimated that the viral concentration was ~10^4^ viruses/ml based on the bacterial cell counts published previously (Zhang et al., 2017) with the general rule that viral count is 10 times of the bacterial count (Maranger and Bird, 1995). Additionally, spacers detected in the BC.3.64 and FW.3.37 genomes recovered here and *Lg. pneumophila* 2300/99 Alcoy matched to contigs in marine metagenomes (AACY02; Venter et al., 2004). Although the matches are not perfect (except one) to organisms in WWTPs or marine environment, the evolving nature of spacers by mutations at CRISPR loci allows us to speculate that WWTPs and marine environments were possible sources of these spacers. Those *Legionella* strains could have come from water bodies under the influence of wastewater or seawater, such as flooded sewers or coastal groundwater.

### Amoebae as a “hub” connecting viruses and intracellular bacteria

This study observed that the prophage exhibiting high sequence similarity to *Pandoravirus* could co-exist with *Acanthamoeba* spp., *Parachlamydia* spp., *Legionella* spp., and *Mycobacterium* spp. in the FW sample. So far, free-living amoebae in drinking water systems are reported to be an ideal shelter to provide nutritional requirements for the growth of *Legionella* (Breiman et al., 1990; Dupuy et al., 2016), and are the only reported host of *Pandoravirus* (Philippe et al., 2013). Various giant viruses, including *Mimivirus, Mamavirus*, and *Pandoravirus*, have been detected in amoebae and were reported to be involved in lateral gene transfer between viruses and bacteria (La Scola et al., 2003, 2008; Philippe et al., 2013). While the detection of *Parachlamydia* in drinking water systems is rare (Thomas et al., 2008), previous studies have suggested that Chlamydiae were likely prevalent in aquatic environments (Barret et al., 2013; Lagkouvardos et al., 2014). These observations all support amoebae as the “hub” connecting viruses and intracellular bacteria, and facilitating the genetic exchange between pathogens and their closely related species (Gimenez et al., 2011; Gomez-Valero et al., 2011). Thus, developing control strategies to eukaryotic populations, e.g., filtration with 1 μm membranes, whose size is larger than bacteria but smaller than amoebae, could be an effective means to suppress the growth and spreading of pathogens in DWDSs (Wadowsky et al., 1988).

In summary, our study demonstrates that metagenomics analysis can be used to determine the presence of VFs in potential pathogens in drinking water production and distribution systems. Future studies combining microbiological, genomic, and metabolic methods at the level of gene expression, protein function and regulation, and bacteria-host interaction can help determine the relationship between the presence of these VFs and pathogenicity in immunocompromised individuals, especially for environmental strains recovered from drinking water systems. Furthermore, the development of genomics analysis can serve as a new platform for the detection, strain typing, and monitoring of pathogens, which can provide novel insights into the surveillance and control of waterborne or water-based pathogens. Characteristic regions in bacterial genomes, such as CRISPR-Cas studied here, can be used in combination with the traditional biomarkers to facilitate and simplify the subtyping of pathogens of potential concern and monitor the distribution of the same strains across different environmental niches.

## Author contributions

YZ designed and carried out the experiments, analyzed the obtained data, and wrote the manuscript. MK and AW carried out the experiments to quantify the pathogens and participated in the manuscript writing process. WL designed and carried out the experiments, analyzed the obtained data, and revised the manuscript.

### Conflict of interest statement

The authors declare that the research was conducted in the absence of any commercial or financial relationships that could be construed as a potential conflict of interest.
