# Supplementary material for: Benefits of Genomic Insights and CRISPR-Cas Signatures to Monitor Potential Pathogens across Drinking Water Production and Distribution Systems
Source: Front Microbiol. 2017 Oct 19;8:2036. doi: 10.3389/fmicb.2017.02036 (PMC5654357; doi:10.3389/fmicb.2017.02036)
Supplement: Supplementary file 1 [file Table1.DOCX]

**Supplementary Information**

**Benefits of genomic insights and CRISPR-Cas signatures to track potential pathogens across drinking water production and distribution systems**

Ya Zhang^1^, Masaaki Kitajima^2^, Andrew J. Whittle^3^, and Wen-Tso Liu^1*^

^1^Department of Civil and Environmental Engineering, University of Illinois at Urbana-Champaign, 205 N. Mathews Ave., Urbana, IL 61810, USA

^2^Division of Environmental Engineering, Faculty of Engineering, Hokkaido University, North 13 West 8, Kita-ku, Sapporo, Hokkaido 060-8628, Japan

^3^Department of Civil and Environmental Engineering, Massachusetts Institute of Technology, Cambridge, MA 02139, USA

**Table S1.** Primer sets and reaction conditions used in ddPCR/real-time PCR.

| Format*^a^* | Target organism | Primer/Probe | Sequence (5'-3') | Gene target | Reference |
| --- | --- | --- | --- | --- | --- |
| ddPCR | *Legionella* spp.*^b^* | PanLeg-F | GGCGACCTGGCTTC | *ssrA* | (Benitez and Winchell, 2013) |
|  |  | PanLeg-R1 | GGTCATCGTTTGCATTTATATTTA |  |  |
|  |  | PanLeg-P1 | FAM-ACGTGGGTTGCAA-MGB-NFQ |  |  |
|  |  |  |  |  |  |
|  | *L. pneumophila ^b^* | Lp-F | TTGTCTTATAGCATTGGTGCCG | *mip* |  |
|  |  | Lp-R | CCAATTGAGCGCCACTCATAG |  |  |
|  |  | Lp-P | HEX-CGGAAGCAA/ZEN/TGGCTAAAGGCATGCA-BHQ1 |  |  |
|  |  |  |  |  |  |
|  | *P. aeruginosa* | Ps-F | CGAGTACAACATGGCTCTGG | *opr*L | (Feizabadi et al., 2010) |
|  |  | Ps-R | ACCGGACGCTCTTTACCATA |  |  |
|  |  | Ps-P | FAM-CCTGCAGCA/ZEN/CCAGGTAGCGC-BHQ1 |  |  |
|  |  |  |  |  |  |
|  | *A. hydrophila* | Ahyd-F | GGCCTTGCGCGATTGTATAT | 16S rRNA | (Trakhna et al., 2009) |
|  |  | Ahyd-R | GTGGCGGATCATCTTCTCAGA |  |  |
|  |  | Ahyd-P | FAM-CAGGTGGGA/ZEN/TTATCTAGTTG-BHQ1 |  |  |
|  |  |  |  |  |  |
|  | *Mycobacterium* spp.*^c^* | My-65kDaf2 | TAGGTCGGGACGGTGAG | 65kDa hsp | (Tobler et al., 2006) |
|  |  | My-65kDar3 | TTGCGAAGTGATTCCTCC |  |  |
|  |  | My-Genus | FAM-CGTCCGTCG/ZEN/CGGGCRCTGMRC-BHQ1 |  |  |
|  |  |  |  |  |  |
|  | *M. tuberculosis* complex*^c^* | Mtbc-complex | HEX-AGTTGTCCT/ZEN/CGCTGCCACTCGCT-BHQ1 |  |  |
|  |  |  |  |  |  |
|  | *Bacteria* universal | BactQuant-F | CCTACGGGDGGCWGCA | 16S rRNA | (Liu et al., 2012) |
|  |  | BactQuant-R | GGACTACHVGGGTMTCTAATC |  |  |
|  |  | BactQuant-TP | FAM-CAGCAGCCGCGGTA-MGB-NFQ |  |  |
|  |  |  |  |  |  |
|  | *Archaea* universal | ARC787F | ATTAGATACCCSBGTAGTCC | 16S rRNA | (Yu et al., 2005) |
|  |  | ARC1059R | GCCATGCACCWCCTCT |  |  |
|  |  | ARC915F-TP | FAM-AGGAATTGG/ZEN/CGGGGGAGCAC-BHQ1 |  |  |
|  |  |  |  |  |  |
| Real-time PCR | *Naegleria fowleri* | JBVF | AGGTACTTACGTTAGAGTGCTAGT | ITS | (Mull et al., 2013) |
|  |  | JBVR | ATGGGACAATCCGGTTTTCTCA |  |  |
|  |  | JBVP | FAM-ACGCCCTAG/ZEN/CTGGTTATGCCGGATT-BHQ1 |  |  |
|  |  |  |  |  |  |
|  | *Acanthamoeba ^d^* | AcantF900 | CCCAGATCGTTTACCGTGAA | 18S rRNA | (Qvarnstrom et al., 2006) |
|  |  | AcantR1100 | TAAATATTAATGCCCCCAACTATCC |  |  |
|  |  | AcantP1000 | FAM-CTGCCACCG/ZEN/AATACATTAGCATGG-BHQ1 |  |  |
|  |  |  |  |  |  |
|  | *B. mandrillaris ^d^* | BalaF1451 | TAACCTGCTAAATAGTCATGCCAAT | 18S rRNA |  |
|  |  | BalaR1621 | CAAACTTCCCTCGGCTAATCA |  |  |
|  |  | BalaP1582 | HEX-AGTACTTCT/ZEN/ACCAATCCAACCGCCA-BHQ1 |  |  |

*^a^* ddPCR allows for the quantification of target genes with higher accuracy for low copy number samples, whereas real-time PCR has been more widely used and provides more reliable validations for the presence/absence of pathogens. For this reason we used real-time PCR for pathogens with greater societal impact, such as *N. fowleri* and other amoebic pathogens.

*^b, c, d^* duplex reactions.

**Table S2.** Quantification results from ddPCR.

|  | ddPCR (copies/ng-gDNA) | | | | | | | |
| --- | --- | --- | --- | --- | --- | --- | --- | --- |
|  | *Bacteria* 16S | *Archaea* 16S | *Mycobacterium* spp. | M. *tuberculosis*-complex | *Legionella* spp. | *L. pneumophila* | *P. aeruginosa* | *A. hydrophila* |
| RW | 841014 | 19654 | 48.4 | - | - | - | - | - |
| BC | 135455 | 1.5 | 38.2 | - | 40.9 | - | - | - |
| FW | 93243 | 3.6 | 16.4 | - | 0.2 | - | 0.1 | - |
| DS1 | N/A | N/A | N/A | N/A | N/A | N/A | N/A | N/A |
| DS2 | 1234637 | 14.9 | 391.0 | - | - | - | - | - |
| DS3 | N/A | N/A | N/A | N/A | N/A | N/A | N/A | N/A |
| PB1 | 55203 | 4.7 | 439.0 | - | 25.7 | - | - | - |
| PB2 | 106376 | - | 36.9 | - | - | - | - | - |
| WM | 8344 | - | 16.6 | - | - | - | - | - |
| PR | 107609 | 40.4 | 33385 | - | - | - | - | - |

-: not detected

N/A: data not available

**Table S3.** Genomes included in Figure 3.

| **Strains** | **Accession number** |
| --- | --- |
| *Legionella longbeachae* NSW150 | FN650140 |
| *Legionella pneumophila* str. Paris | NC_006368 |
| *Legionella fairfieldensis* ATCC 49588 | JHYC01000001 |
| *Mycobacterium tuberculosis* H37Rv | NC_000962 |
| *Mycobacterium gordonae* CTRI 14-8773 | LKTM01000001 |
| *Mycobacterium avium* subsp. paratuberculosis K-10 | NC_002944 |
| *Mycobacterium chelonae* strain ATCC 35752 | CP010946 |
| *Leptospira interrogans* serovar Lar str. 56601 | NC_004342 |
| *Leptospira borgpetersenii* serovar Hardjo-bovis str. L550 | NC_008508 |
| *Leptospira biflexa* serovar Patoc strain 'Patoc 1 (Paris)' | NC_010602 |
| *Parachlamydia acanthamoebae* UV-7 | NC_015702 |
| *Candidatus* Protochlamydia amoebophila UWE25 | NC_005861 |

**Table S4.** Detected antibiotic resistance genes in the recovered draft genomes.

| **Affiliations** | **Genomes** | **Resistance gene** | **%Identity** | **Predicted phenotype** |
| --- | --- | --- | --- | --- |
| *Legionella* spp. | DS3.009 | aph(9)-la | 84 | Aminoglycoside resistance |
|  |  | blaOXA-29 | 84 | Beta-lactam resistance |
|  |  | cpxR | 88 | Resistance to cefepime, chloramphenicol, antimicrobial peptides |
| *Mycobacterium* spp. | DS1.3.26,  DS2.013,  PR.002 | aac(2')-Ic | 82 | Aminoglycoside resistance |

**Table S5.** *cas* gene similarities among *Legionella* spp.

|  | *cas1* | *cas3* | *csy1* | *csy2* | *csy3* | *csy4* |
| --- | --- | --- | --- | --- | --- | --- |
| BC.3.64/FW.3.37 | 99% | 100% | 100% | 100% | 100% | 100% |
| BC.3.64/Alcoys | 74% | 74% | <40% | <40% | <40% | 50% |
| BC.3.64/Lens.chromosome | 76% | 51% | 48% | 51% | 65% | 49% |
| Lens.chromosome/Lens.plasmid | 99% | 98% | 97% | 96% | 99% | 96% |
| Alcoys/Len.chromosome | 84% | <40% | <40% | <40% | <40% | 64% |

**Supplementary figure legends**

**Figure S1.** An illustration of sampling sites and events for the studied drinking water system. Water-phase samples included RW, BC, FW, and DS1-3. Biofilm phase samples included PB1, PB2, WM, and PR. Each open dot represents one sampling event.

**Figure S2.** Detected *ntt* genes with BC.030. Sequences of PamNTT1-5 were from a previous report (Haferkamp et al., 2006).

**Figure S3.** Detected CRISPR-Cas locus in FW.030.

**Figure S4.** Putative prophage sequences in the recovered draft genomes. Pa: *Parachlamydia*; Lp: *Leptospira*.

**Figure S5.** Prophage sequences in the retrieved draft genomes with integrases. Pa: *Parachlamydia*; Lp: *Leptospira*. Shaded regions represented highly similar prophages.

**Figure S6.** The neighbor-joining tree for the *mip* gene.


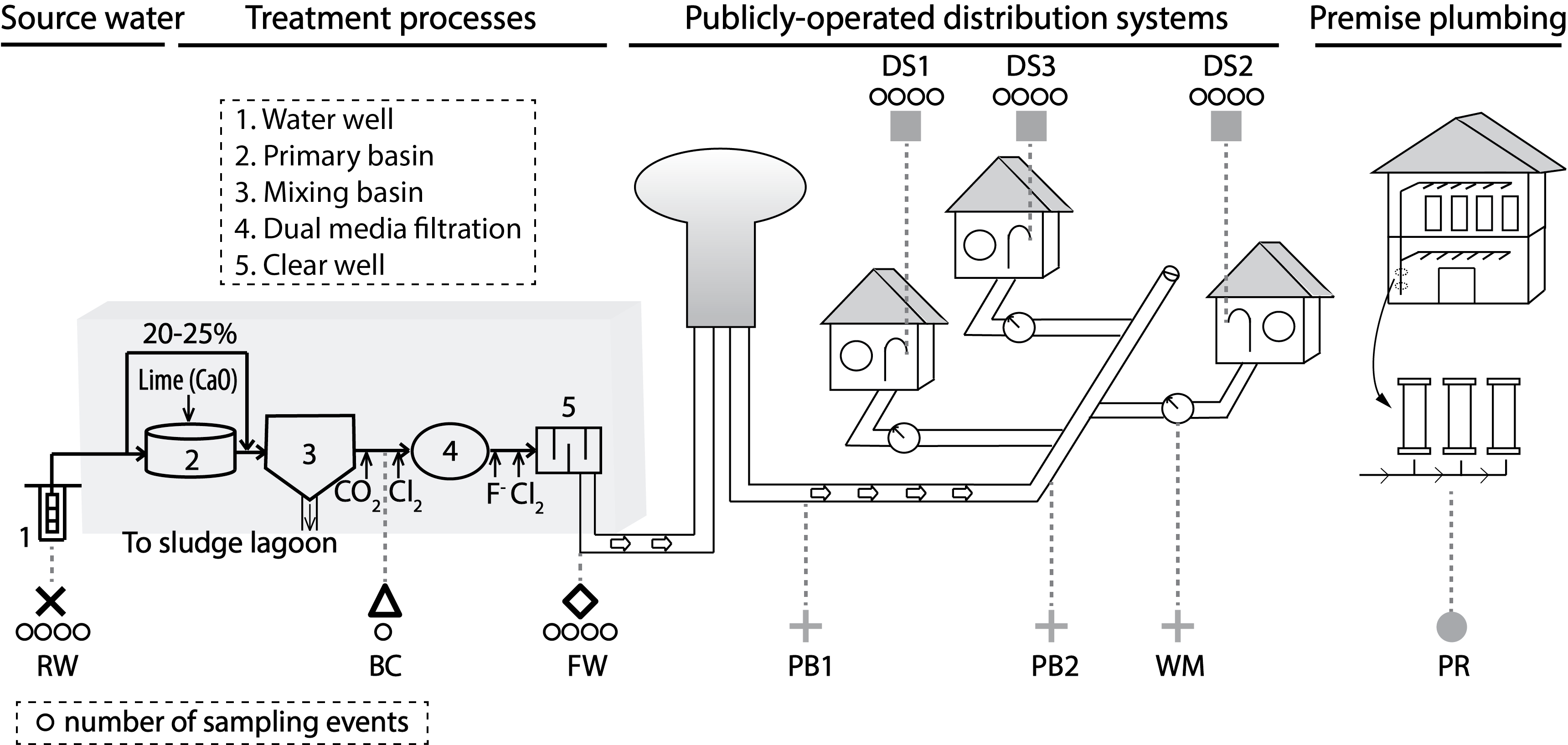


**Figure S1.** An illustration of sampling sites and events for the studied drinking water system. Water-phase samples included RW, BC, FW, and DS1-3. Biofilm phase samples included PB1, PB2, WM, and PR. Each open dot represents one sampling event.

**
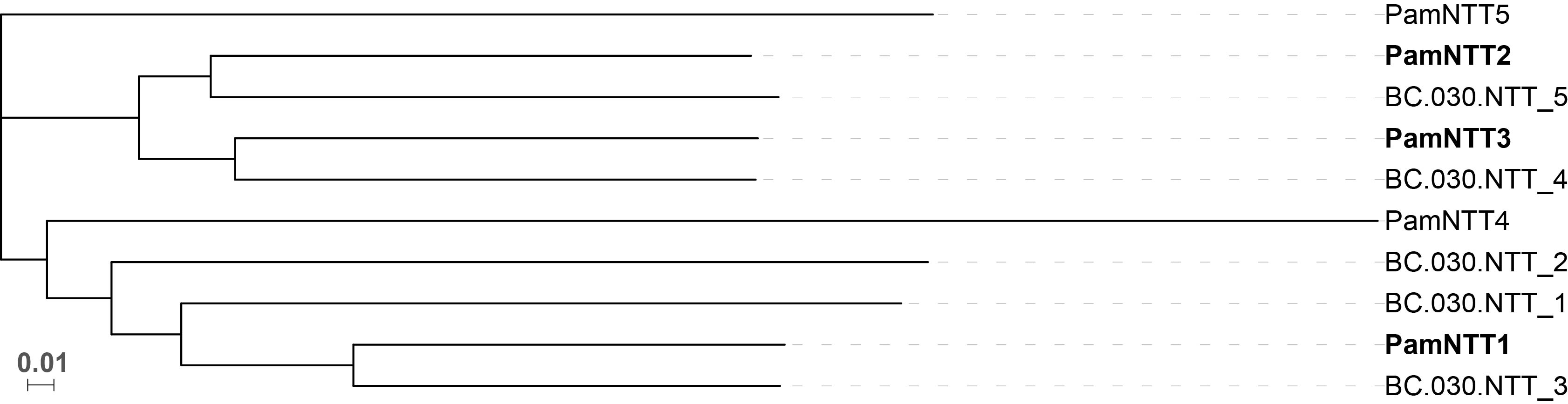
Figure S2.** Detected *ntt* genes with BC.030. Sequences of PamNTT1-5 were from a previous report (Haferkamp et al., 2006).


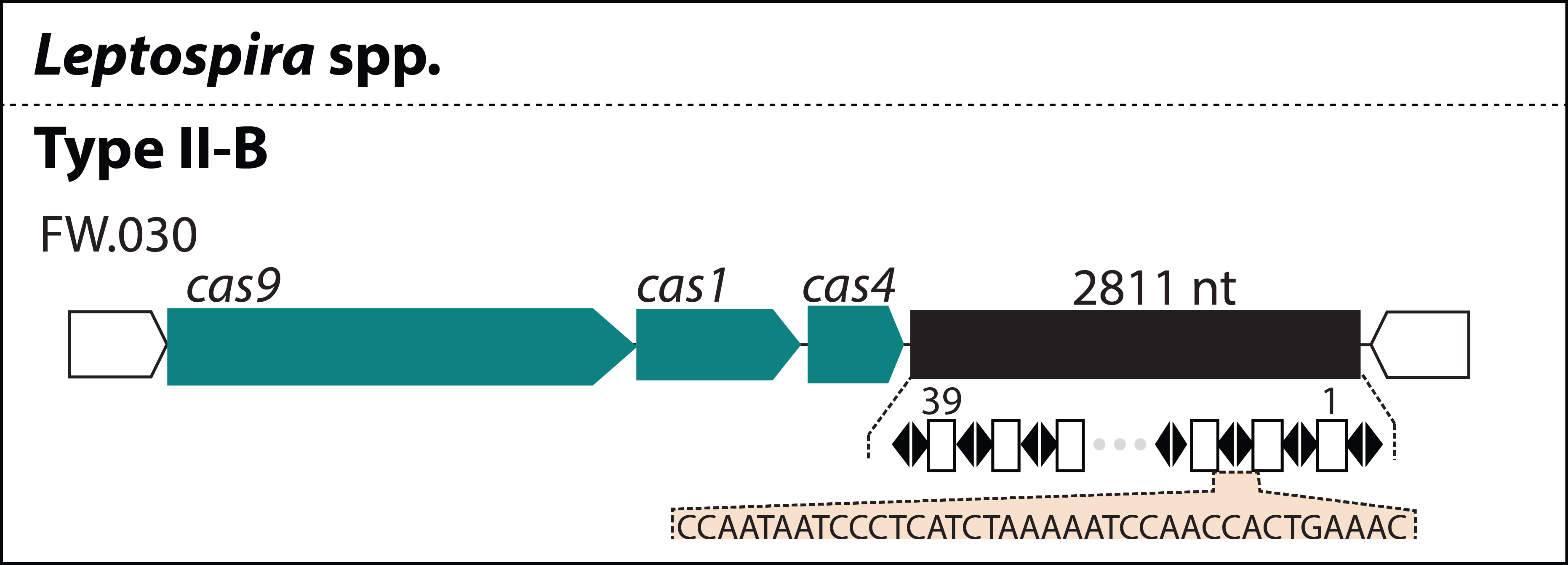


**Figure S3.** Detected CRISPR-Cas locus in FW.030.


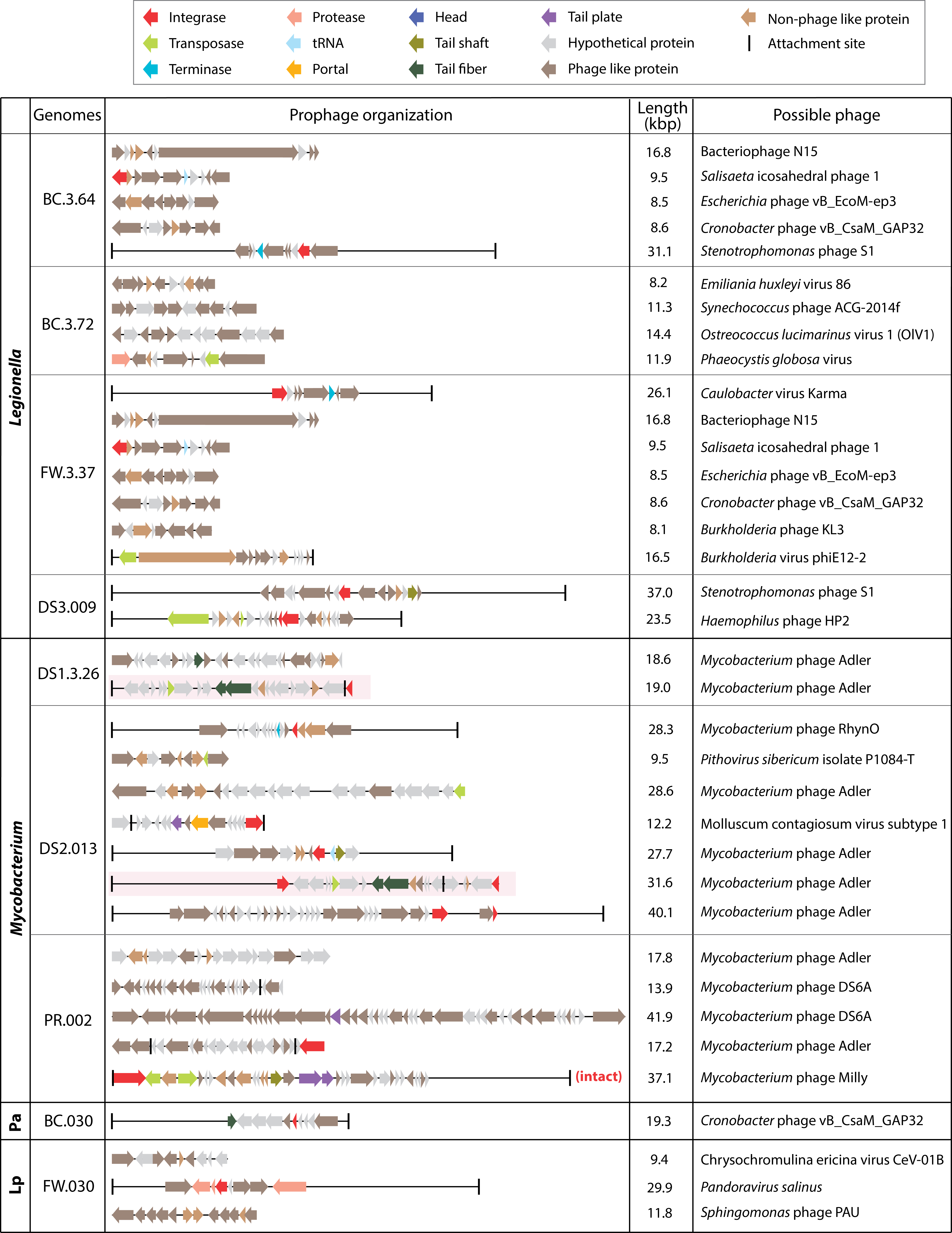


**Figure S4.** Putative prophage sequences in the recovered draft genomes. Pa: *Parachlamydia*; Lp: *Leptospira*.


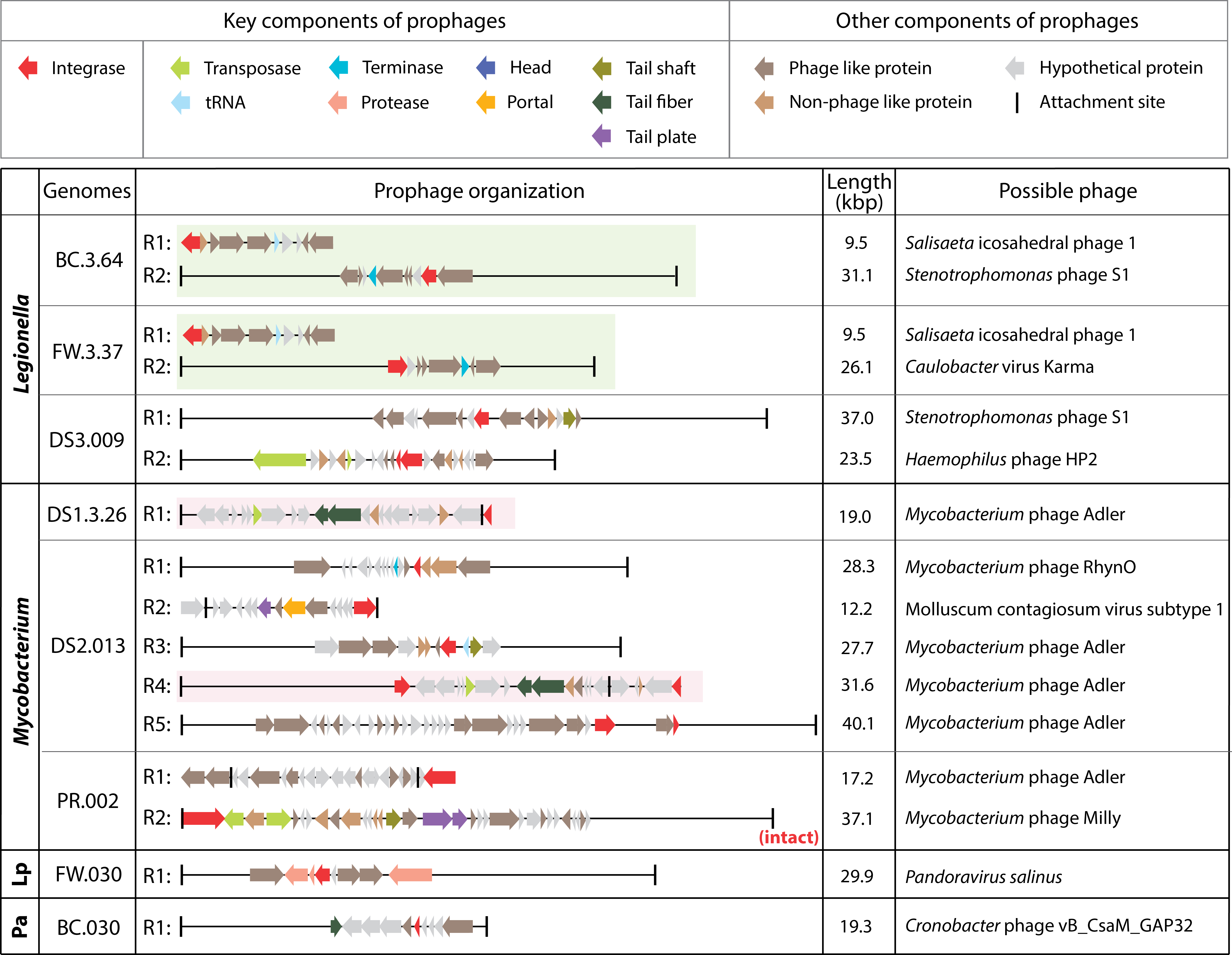


**Figure S5.** Prophage sequences in the retrieved draft genomes with integrases. Pa: *Parachlamydia*; Lp: *Leptospira*. Shaded regions represented highly similar prophages.

**
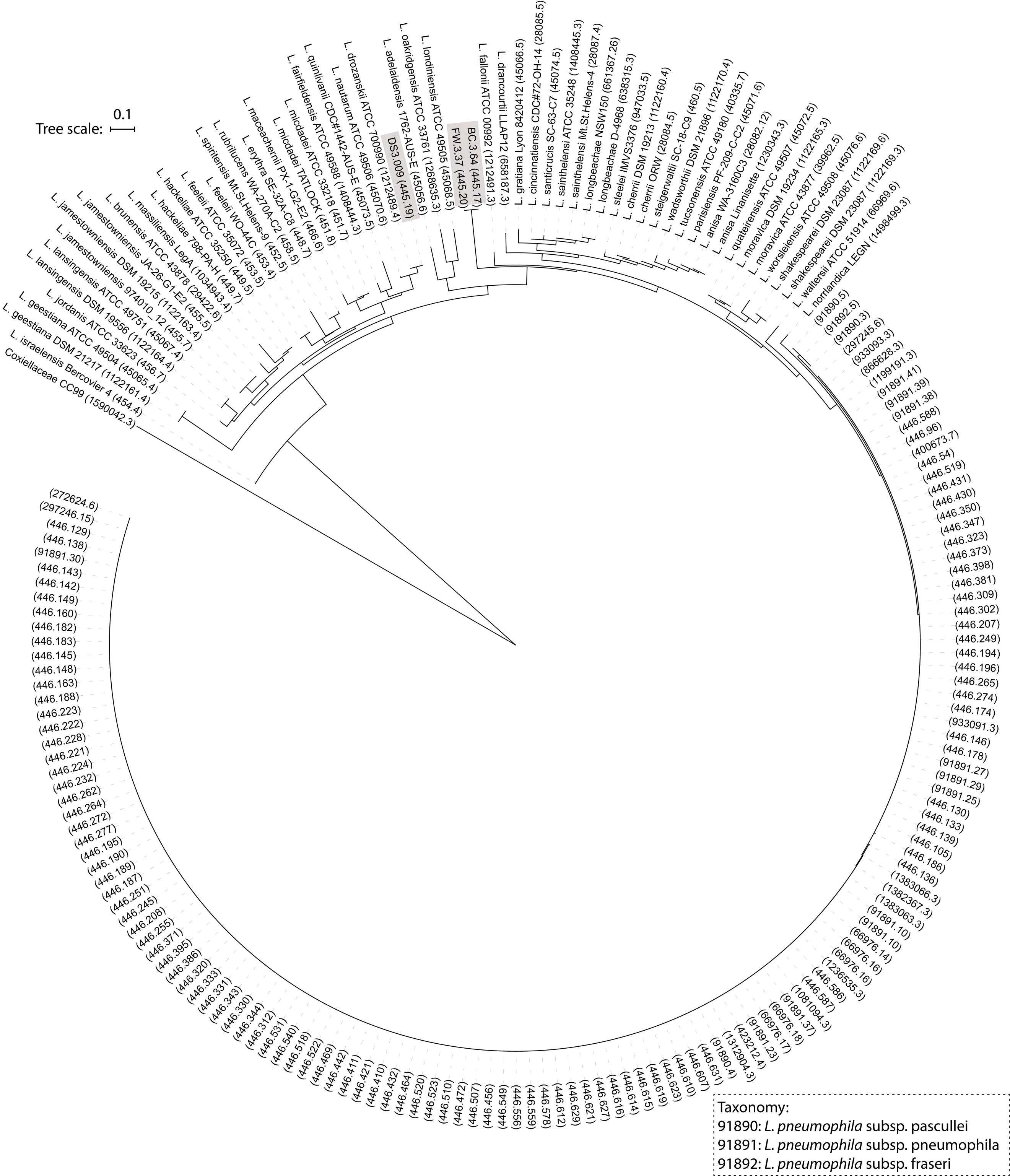
**

**Figure S6.** The neighbor-joining tree for the *mip* gene.

**References:**

Benitez, A.J., and Winchell, J.M. (2013). Clinical application of a multiplex real-time PCR assay for simultaneous detection of Legionella species, Legionella pneumophila, and Legionella pneumophila serogroup 1. *J Clin Microbiol* 51(1)**,** 348-351. doi: 10.1128/JCM.02510-12.

Feizabadi, M.M., Majnooni, A., Nomanpour, B., Fatolahzadeh, B., Raji, N., Delfani, S., et al. (2010). Direct detection of Pseudomonas aeruginosa from patients with healthcare associated pneumonia by real time PCR. *Infect Genet Evol* 10(8)**,** 1247-1251. doi: 10.1016/j.meegid.2010.08.008.

Haferkamp, I., Schmitz-Esser, S., Wagner, M., Neigel, N., Horn, M., and Neuhaus, H.E. (2006). Tapping the nucleotide pool of the host: novel nucleotide carrier proteins of Protochlamydia amoebophila. *Mol Microbiol* 60(6)**,** 1534-1545. doi: 10.1111/j.1365-2958.2006.05193.x.

Liu, C.M., Aziz, M., Kachur, S., Hsueh, P.R., Huang, Y.T., Keim, P., et al. (2012). BactQuant: An enhanced broad-coverage bacterial quantitative real-time PCR assay. *BMC Microbiol* 12. doi: 10.1186/1471-2180-12-56.

Mull, B.J., Narayanan, J., and Hill, V.R. (2013). Improved Method for the Detection and Quantification of Naegleria fowleri in Water and Sediment Using Immunomagnetic Separation and Real-Time PCR. *J Parasitol Res* 2013**,** 8. doi: 10.1155/2013/608367.

Qvarnstrom, Y., Visvesvara, G.S., Sriram, R., and da Silva, A.J. (2006). Multiplex real-time PCR assay for simultaneous detection of Acanthamoeba spp., Balamuthia mandrillaris, and Naegleria fowleri. *J Clin Microbiol* 44(10)**,** 3589-3595. doi: 10.1128/JCM.00875-06.

Tobler, N.E., Pfunder, M., Herzog, K., Frey, J.E., and Altwegg, M. (2006). Rapid detection and species identification of Mycobacterium spp. using real-time PCR and DNA-Microarray. *J Microbiol Methods* 66(1)**,** 116-124. doi: 10.1016/j.mimet.2005.10.016.

Trakhna, F., Harf-Monteil, C., Abdelnour, A., Maaroufi, A., and Gadonna-Widehem, P. (2009). Rapid Aeromonas hydrophila identification by TaqMan PCR assay: comparison with a phenotypic method. *Lett Appl Microbiol* 49(2)**,** 186-190. doi: 10.1111/j.1472-765X.2009.02635.x.

Yu, Y., Lee, C., Kim, J., and Hwang, S. (2005). Group-specific primer and probe sets to detect methanogenic communities using quantitative real-time polymerase chain reaction. *Biotechnol Bioeng* 89(6)**,** 670-679. doi: 10.1002/bit.20347.
